# Validation of a continuous infusion of low dose Iohexol to measure glomerular filtration rate: randomised clinical trial

**DOI:** 10.1186/s12967-015-0414-3

**Published:** 2015-02-12

**Authors:** John J Dixon, Katie Lane, R Neil Dalton, Charles Turner, R Michael Grounds, Iain AM MacPhee, Barbara J Philips

**Affiliations:** General Intensive Care Unit, St. George’s Hospital, London, UK; Acute Kidney Injury Research Group, Division of Clinical Sciences, St. George’s, University of London, London, UK; WellChild Laboratory, King’s College London, Evelina Children’s Hospital, London, UK; Renal Medicine, St. George’s Hospital, London, UK

**Keywords:** Acute kidney injury, Glomerular filtration rate, Iohexol, Validation

## Abstract

**Introduction:**

There is currently no accurate method of measuring glomerular filtration rate (GFR) during acute kidney injury (AKI). Knowledge of how much GFR varies in stable subjects is necessary before changes in GFR can be attributed to AKI. We have designed a method of continuous measurement of GFR intended as a research tool to time effects of AKI. The aims of this crossover trial were to establish accuracy and precision of a continuous infusion of low dose Iohexol (CILDI) and variation in GFR in stable volunteers over a range of estimated GFR (23-138 mL/min/1.73 m^2^).

**Methods:**

We randomised 17 volunteers to GFR measurement by plasma clearance (PC) and renal clearance (RC) of either a single bolus of Iohexol (SBI; routine method), or of a continuous infusion of low dose Iohexol (CILDI; experimental method) at 0.5 mL/h for 12 h. GFR was measured by the alternative method after a washout period (4–28 days). Iohexol concentration was measured by high performance liquid chromatography/electrospray tandem mass spectrometry and time to steady state concentration (*Css*) determined.

**Results:**

Mean PC was 76.7 ± 28.5 mL/min/1.73 m^2^ (SBI), and 78.9 ± 28.6 mL/min/1.73 m^2^ (CILDI), p = 0.82. No crossover effects occurred (p = 0.85). Correlation (*r*) between the methods was 0.98 (p < 0.0001). Bias was 2.2 mL/min/1.73 m^2^ (limits of agreement −8.2 to 12.6 mL/min/1.73 m^2^) for CILDI. PC overestimated RC by 7.1 ± 7.3 mL/min/1.73 m^2^. Mean intra-individual variation in GFR (CILDI) was 10.3% (p < 0.003). Mean ± SD *Css* was 172 ± 185 min.

**Conclusion:**

We hypothesise that changes in GFR >10.3% depict evolving AKI. If this were applicable to AKI, this is less than the 50% change in serum creatinine currently required to define AKI. CILDI is now ready for testing in patients with AKI.

**Trial registration:**

This trial was registered with the European Union Clinical Trials Register (https://www.clinicaltrialsregister.eu/), registration number: 2010-019933-89.

## Introduction

The absence of an accurate method of measuring changing glomerular filtration rate (GFR) in acute kidney injury (AKI) poses a significant barrier to research in this area. Identifying when pathophysiological changes associated with AKI occur remains a significant challenge. Current definitions of AKI are based upon an increase in serum creatinine concentration (SCr) of greater than 50% or reduction in urine output (UO) [1-3] despite limitations in interpretation of these parameters in critically ill patients [4]. Changes in SCr sufficient to define AKI may be delayed, particularly in patients with chronic kidney disease (CKD) [5] or sepsis [6]. Acute illness may lead to diminished creatinine formation [7] limiting its utility as a biomarker for GFR in AKI. Other endogenous biomarkers have been investigated but have variable performance [8]. None have been proven to be superior to SCr and UO in heterogenous populations (e.g. general critical care units), or where the onset of the insult or its aetiology is unclear.

Using exogenous markers, such as radioactive ethylene diamine tetraacetic acid (EDTA) or radio-opaque contrast media, to measure GFR in AKI have advantages over endogenous markers: they are not influenced by body habitus, diet or metabolic processes, and are not dependent on the timing of the insult causing AKI. Exogenous markers can theoretically be administered as a single bolus injection and measuring the time to elimination from the body, or as a continuous infusion. Single bolus injection of Iohexol (SBI) has been used in critically ill patients [9], but interpretation of this approach assumes stable GFR, which is unlikely in the context of evolving AKI: administration would need to be repeated frequently to track changing GFR, and may lead to accumulation of Iohexol. Furthermore, bolus methods are inappropriate in AKI because of the washout period required.

Continuous infusions require stable GFR and volume of distribution (*Vd*) to achieve steady state concentration (*Css*). They are limited by natural intra-individual variations in GFR (*precision*) and bias of laboratory analytical equipment. *Css* varies between patients according to: a) baseline GFR, b) *Vd*, and c) the mass of substance infused over time (*Minf*). In stable patients (a) and (b) are unchanged, and (c) is controlled by the operator. *Css* can be predicted if the subject’s weight and baseline GFR are known. Once *Css* has been reached, variations in GFR occurring after this time, in excess of precision and bias, likely represent true changes in GFR. Theoretically, measurements made after time to *Css* will represent GFR at that moment. A loading dose (LD) given prior to the infusion reduces time to *Css*: if LD is too large, plasma concentration declines until *Css* is reached; if too small, plasma concentrations climb until *Css* is reached. In AKI, *Css* may never be reached, however, plasma measurements made after the predicted time to *Css* can detect changes from baseline GFR and predicted *Css*. In rapidly evolving AKI occurring before time to *Css*, concentrations will not change towards *Css* at the expected rate, and may even increase. To date, there have been no studies measuring changing GFR in AKI using continuous infusions.

Administration of a continuous infusion of low dose Iohexol (CILDI; Omnipaque 300®, at 0.5 mL/h) has the potential to measure evolving GFR in AKI. A continuous infusion of Iohexol has previously been validated in subjects with normal GFR [10], however, *Css* and time to *Css* in subjects with CKD are unknown: prolonged time to *Css* in patients with CKD would limit the applicability of CILDI in patients with AKI.

We have performed a proof-of-method clinical trial (randomised crossover design) with the aim of validating CILDI for measuring GFR as a research tool in AKI. CILDI was compared to measurement of the plasma clearance (PC) of a SBI [9,11]. We have used the SBI method as our “gold standard”, rather than a continuous infusion for three reasons: 1) The SBI method has previously been validated to measure a wide range of GFR in stable patients, from normal to measuring residual renal function in patients requiring renal replacement therapy [11,12]. We are therefore confident that the SBI method is accurate and precise. 2) The SBI method previously used in critically ill patients [9] may be regarded by some authors as a “gold standard”, however, we think this is inappropriate in AKI for reasons listed above. We wanted to demonstrate that CILDI is not inferior to the SBI method when measuring GFR in stable patients; 3) No continuous infusion has been tested in AKI and after proof of methods our intention is to test the method in such patients. Urine was collected, so that Iohexol renal clearance (RC) could be measured. Healthy volunteers (HV; defined as estimated GFR >60 mL/min/1.73 m^2^ by the simplified MDRD equation [13]) and patients with stable CKD (defined as eGFR <60 mL/min/1.73 m^2^) were recruited so that the variability of GFR in stable patients using our method could be determined over a wide range of GFR equivalent to that expected to occur in patients developing AKI. The range of eGFR in the HV cohort was 75-138 mL/min/1.73 m^2^, and the eGFR range in the CKD cohort was 23-59 mL/min/1.73 m^2^.

### Objectives

1) Compare the performance of CILDI with the SBI method in HV and patients with stable CKD. 2) Measure intra-individual variation of GFR in stable subjects, so that the minimum change in GFR (precision) detected by CILDI can be determined. 3) Confirm that subjects with eGFR >23 mL/min/1.73 m^2^ have time to *Css* <12 hours.

This article represents the first stage: validation of the technique and establishment of the accuracy and precision in subjects with stable GFR. The goal is to use CILDI in patients with, and at risk of, developing AKI. Changing GFR associated with AKI can be measured by PC and RC at various time points after predicted time to *Css*, allowing the temporal relationship between AKI and its pathophysiological effects to be delineated.

## Methods

### Clinical trial registration and ethics

This trial was registered with the European Union Clinical Trials Register (https://www.clinicaltrialsregister.eu), registration number: 2010-019933-89. Approval was obtained from Brighton East Research Ethics Committee (Ref: 10/H1107/24). The Declaration of Helsinki (2008) [14] was adhered to throughout. All subjects provided prior written informed consent. The trial was sponsored by St. George’s, University of London, United Kingdom.

### Setting

The trial took place in the Clinical Research Facility, St. George’s, University of London. Research methods were performed to International Conference for Harmonisation Good Clinical Practice standards [15].

### Recruitment, inclusions and exclusions

Subjects were recruited from local Nephrology outpatient clinics or via advertisements placed on public notice boards within St. George’s Healthcare NHS Trust or St. George’s, University of London.

#### Inclusions

Adults aged 18–75 years with renal function ranging from normal to chronic kidney disease (CKD) stage 4. Subjects were classified as having CKD if their estimated GFR was <60 mL/min/1.73 m^2^ by the simplified MDRD equation [15] or healthy volunteers (HV) if eGFR was >60 mL/min/1.73 m^2^.

#### Exclusions

These were precautionary and based on the listed criteria for radio-opaque contrast media [16]. Reactions to radio-contrast media; thyroid disease, myasthenia gravis, cardiac arrhythmias, pulmonary hypertension, epilepsy, structural brain disease, phaeochromocytoma, advanced heart failure, sickle cell disease, multiple myeloma, homocystinuria, ascites, pregnancy or breast-feeding, renal replacement therapy; subjects taking Metformin if serum creatinine >150 μmol/L, Phenothiazines, Tricyclic antidepressants, Monoamine oxidase inhibitors, Levo-thyroxine, Amiodarone, Interleukin-2 agents; a planned Tc99m–labelled scan. Subjects unable to provide written Informed Consent were also excluded.

### Study design and randomisation

Computer-generated block randomisation allocated subjects to measurement of GFR via Method A (plasma clearance of a single bolus of Iohexol; SBI), or Method B (continuous infusion of low dose Iohexol; CILDI). Subjects then underwent a washout period of 4–28 days before GFR was measured by the alternative method. Four days was chosen as the minimum washout period because we wanted to ensure that subjects entering the second part of the crossover study had no remaining Iohexol within their body, and 4 days is more than double the time for Iohexol to be completely eliminated in a subject with GFR <20 mL/min/1.73 m^2^ [11,16]. An epidemiological study has suggested the rate of progression of CKD in stable subjects may be as high as a loss in GFR of 3.1 mL/min/1.73 m^2^ per year [17], so 28 days was chosen as the maximum washout period to minimise the likely risk of GFR changing between the two methods. A crossover design was used to further mitigate potential bias caused by changing clinical conditions.

### Iohexol administration and sampling

*Procedures common to both methods:* body surface area (BSA) was calculated from height and weight [18]. An intravenous cannula was inserted into each arm. Iohexol was administered via one cannula and 2 mL blood samples collected from the other into serum separator containers. Serum was centrifuged at 4°C at 3500 rpm for 10 min. Every time urine was voided, subjects’ bladders were scanned to ensure complete emptying (Bladderscan® BVI9400, Verathon Medical UK Ltd.). Urine and serum samples were stored at −80°C. The CKD group received intravenous 1.4% Sodium Bicarbonate 100 mL/h, in accordance with local hospital guidelines. Healthy volunteers were encouraged to drink 100 mL/h water. Volunteers were allowed to eat and drink freely.

#### Method A (SBI)

5 mL Iohexol (Ominpaque 300®) was administered as an intravenous bolus over 2 minutes [9]. The first blood sample was collected at 5 minutes. This allowed later confirmation that intravenous administration had occurred (a high concentration at 5 min suggests intravenous, rather than subcutaneous, injection). Samples were collected at 2 h, 3 h, and 4 h to calculate GFR. Urine was taken to measure renal clearance twice.

#### Method B (CILDI)

An intravenous loading dose (LD) was administered according to the formula:

$$ LD= volume\kern0.5em  of\kern0.5em  distribution\;(Vd)\kern0.5em \times \kern0.5em  target\kern0.5em  steady\kern0.5em  state\kern0.5em  concentration\kern0.5em (Css) $$

From the Summary of Product Characteristics [16], Vd = 0.165 L/Kg (95% CI: 0.108-0.219) × Weight (Kg). The target steady state concentration (100 μmol/L) is approximately 100 times the lower limit of quantitation by high performance liquid chromatography/tandem mass spectrometry (LC-MSMS): the actual steady state achieved in individuals was likely to vary according to their GFR and *Vd*. Ideally the LD would be calculated using GFR, however, weight was used so that CILDI could eventually be used in patients with an unknown baseline GFR. A continuous intravenous infusion of Iohexol (Omnipaque 300®) was then administered at 0.5 mL/h (343.5 mg/mL) for 12 h (Agilia MC Injectomat, Fresenius Kabihas) [19]. Blood samples were taken at 30 min, 60 min, 90 min, 2 h, 3 h, 4 h, 6 h, 8 h, 10 h, and 12 h. Urine samples were collected between each plasma sample, when possible.

### GFR calculation

*Method A*: the natural logarithms of serum Iohexol concentrations at 2 h, 3 h, and 4 h were plotted against time. The intercept and slope were used to derive the theoretical time zero concentration of Iohexol, *Vd* and plasma half-life (*T*_*1/2*_*)*. GFR was calculated by dividing the product Log_e_(2)*Vd by *T*_*1/2*_, adjusting for BSA [18] and applying the Bröchner-Mortensen single compartment correction factor [20]. Renal Clearance (RC) was derived by measuring the urine concentration at 2.5 and 3.5 h and calculating the corresponding plasma concentration at 2.5 h and 3.5 h from the log-concentration-time graph, and using the formula: *GFR*(*mL*/*min*) = [*U* × *V*]/*P*. Where *U* = urine Iohexol concentration (μmol/L), *V* = volume of urine (mL) per unit time (min), and *P* = plasma Iohexol concentration (μmol/L), adjusting for BSA [18]. The mean of the two values was used for RC.

*Method B (CILDI):* results were plotted on a 2-phase exponential decay curve, and the plateau concentration calculated. Plasma clearance was calculated by the formula: *GFR*(*mL*/*min*) = [*Iohexol infusion rate* (*μmol*/*min*)]/[*serum plateau Iohexol concentration* (*μmol*/*mL*)], and adjusting for BSA. Renal clearance was calculated when bladder voidance and urine collection were complete, by measuring urine concentration of Iohexol at the mid-time point between each plasma sample after the time to steady state had been reached. The mean value of a minimum of two measurements was used for each subject.

### Laboratory procedures

The detailed LC-MSMS method for measurement of plasma Iohexol and Creatinine has been published [21]. A brief summary follows: Frozen samples were defrosted at 4°C and centrifuged at 1500 rpm at 4°C for 4 minutes to separate particulate matter. Serum was decanted into 10 μL aliquots. 50 μL of stabilising fluid was added to each aliquot. This consisted of 10 mL de-ionised water, 250 μL D_5_-Iohexol, 25 μL D_3_-Creatinine, 25 μL D_6_-asymmetrical dimethylarginine, and 1.5 μL symmetrical dimethylarginine. 200 μL 1% (vol/vol) acetonitrile (Rathburn Chemicals Ltd., Walkerbrum, Peebleshire, UK) was added to precipitate the mixture, before centrifugation at 20000 rpm for 3 minutes at 4°C. 200 μL of the mixture was transferred into a 96-well polypropylene well plate and analysed by the API 5000LC/msms with QJET Ion guide accelerated by LINAC® collision cell (AB Applied Biosystems MDS SCIEX). Three quality controls were used with known plasma concentrations of Iohexol (10.6 μmol/L, 516.0 μmol/L, and 99.2 μmol/L).

### Accuracy and precision

Tubular Creatinine secretion varies between 10 and 40% [22]. The proportion of secreted Creatinine can be measured by calculating the fractional excretion of creatinine (ie the fraction of filtered creatinine excreted in the urine divided by measured GFR). Values >100% imply additional tubular secretion. Accurate intravenous administration of Iohexol was confirmed by calculating the fractional excretion of Creatinine (*Fe*_*Creat*_), using Iohexol as the substitute for GFR. *Fe*_*Creat*_ >140% implies that the Iohexol was not administered intravenously. Inaccurate results were not analysed further. Accuracy of GFR measurement by CILDI was determined by performing a Bland-Altman comparison [23] against the SBI method.

GFR calculation during single Iohexol bolus administration was deemed precise if the Pearson correlation co-efficient of the log_e_(Iohexol concentration)-time graph was < −0.985 [24]. Precision of CILDI was calculated by measuring co-efficient of variation (CV) and standard deviation of Iohexol measurements at steady state; from this, precision at the 95% and 99% confidence levels and at 3 standard deviations were calculated, allowing mean intra-individual variation in GFR to be determined.

### Sample size calculation

A difference in mean GFR of <10% between the two methods was considered acceptable [11,12]. The intra-individual CV of repeated measurements of Iohexol plasma clearance has been reported as 5.4% [25]. Based on this, a sample size of 30 subjects has 90% power to detect a GFR difference of 4.7 mL/min/1.73 m^2^ from the mean and a sample size of 17 subjects has 82% power. The sample size was revised to 17 subjects following an interim analysis that demonstrated the difference between the means of both methods was actually 3.5% and the trial was stopped early because we had already recruited more than the required number of subjects. A revised power calculation revealed that a sample size of 17 subjects has 90% power to detect a difference between the means of 2.6 mL/min/1.73 m^2^ whereas 30 subjects would have >99% power.

### Statistical methods

A futility analysis was performed to determine whether results would be different if our trial continued until 30 subjects had completed it. Logarithmic transformation of GFR was performed and data were compared using the paired 2-tailed *t*-test, assessing for period effects during this crossover trial. Difference in mean GFR was compared using the *t*-test. Linear regression using Pearson’s correlation was performed to assess association between the methods, and level of agreement was assessed by the Bland Altman comparison [23]. Graphpad Prism®, version 5.0d (Graphpad software, Inc.) was used for statistical analysis. Because the trial was stopped early, all statistical results were reviewed and approved by a statistician independent to our trial.

## Results

### Screening, enrolment and subjects

**Figure 1:**
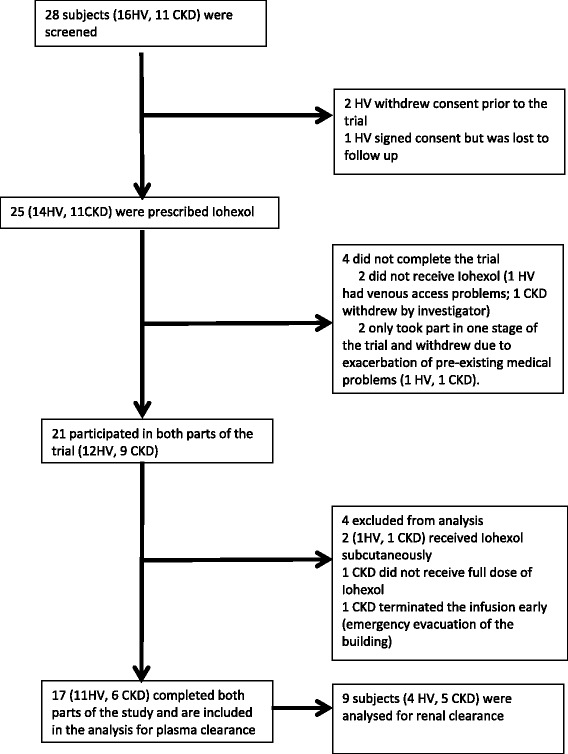
**Subject screening and participation.** No adverse effects due to Iohexol were observed. HV = healthy volunteers; CKD = Patients with chronic kidney disease.

Twenty-one subjects entered both parts of the crossover trial. Four subjects were excluded from full analysis: 3 because of Iohexol administration errors and 1 because of an emergency evacuation of the building during method B. Seventeen subjects completed the trial for the measurement of Iohexol PC. RC was accurately measured in 9 subjects. Details are outlined in Figure 1. Demographic details are summarised in Table 1. Accuracy and precision of Iohexol GFR measurements are listed in Table 2. Laboratory measurements were deemed accurate if the fractional excretion of Creatinine (*Fe*_*Creat*_), using Iohexol as the denominator, was 110-140%; subjects with inaccurate results were excluded. There was no difference in SCr and creatinine clearance in subjects between the two Iohexol GFR study periods (Table 3).

**Table 1:** **Demographic features of trial subjects**

**Subject**	**Age**	**Sex**	**Ethnicity**	**BSA (m** ^**2**^ **)**	eGFR* (mL/min/1.73 m ^**2**^ **)**
*Healthy volunteers*					
1	27	F	AC	1.58	134.9
2	30	F	C	1.75	111
3	20	F	C	1.90	138
4	23	F	C	1.82	98.7
5	28	M	C	2.11	114.5
6	46	F	C	1.69	74.9
7	51	M	C	2.11	87.7
8	35	M	A	1.65	108.7
9	55	F	C	1.74	99.9
10	71	M	C	1.95	100.3
11	49	M	A	2.03	81.1
*Chronic Kidney Disease*					
12	49	F	AC	1.78	34.1
13	74	M	C	2.05	27.2
14	72	M	C	2.00	46.3
15	55	M	C	1.83	59.0
16	51	F	A	1.87	49.4
17	74	M	C	2.00	23.0

BSA = body surface area, F = female, M = male, AC = Afro-Caribbean, C = Caucasian, A = Asian. *eGFR = estimated glomerular filtration rate based upon the simplified MDRD equation^28^. Subjects were classified as healthy volunteers if eGFR > 60 mL/min/1.73 m^2^ and as CKD if eGFR <60 mL/min/1.73 m^2^.

**Table 2:** **Accuracy and precision of Iohexol measurements during methods A (single Iohexol bolus) and B (CILDI)**

**Subject**	**Method A**	**Method B**	**Method A**	**Method B**
	**(FeCreat; %)**	**(FeCreat; %)**	**Precision (Pearson’s r)**	**Precision (CV; %) [no. samples]**
*Healthy Volunteers*				
1	131.7	130.8	−1.00	2.1 [7]
2	132.9	126.0	−1.00	4.4 [4]
3	125.7	122.1	−1.00	1.8 [6]
4	123.7	118.4	−1.00	6.6 [4]
5	117.2	120.4	−0.99	1.8 [7]
6	125.8	122.9	−1.00	2.0 [6]
7	121.8	125.3	−0.99	0.5 [8]
8	133.7	120.3	−0.99	1.6 [5]
9	128.1	128.7	−0.99	3.0 [7]
10	131.5	123.0	−0.97	3.8 [6]
11	121.0	120.4	−0.99	3.2 [6]
Mean	126.6	123.6		2.8 [6]
*Chronic Kidney Disease*				
12	120.7	118.1	−1.00	2.8 [3*]
13	129.8	127.7	−0.99	4.2 [3*]
14	132.8	132.0	−1.00	4.8 [3*]
15	133.4	132.6	−1.00	2.5 [4]
16	133.0	128.4	−1.00	2.2 [3]
17	131.4	133.1	−0.90	2.6 [3*]
Mean	130.2	128.6		3.2 [3]
*All volunteers*				
Mean	127.9	125.5		2.9

Measurements were considered to be accurate if *Fe*_*Creat*_ of 110-140%, using Iohexol as the denominator. Precision in method A is measured by the correlation between the log_e_ Iohexol concentration and time. The measurements are deemed to be precise if: −0.99 ≤ *r* ≥ −1. Correlation is rounded to 2 decimal places. Precision in method B is measured by the co-efficient of variation (CV) of Iohexol concentration measurements once steady state had been achieved, and has been rounded to two significant digits. The number of samples used is in parentheses. In subjects with time to steady state >8 h, the last 3 samples were used (*). *Fe*_*Creat*_ = fractional excretion of Creatinine, *r* = Pearson’s correlation, HV = healthy volunteers, CKD = chronic kidney disease.

**Table 3:** **Comparison of GFR measurements during method A (single Iohexol bolus) and method B (CILDI)**

	**Creatinine**				**Iohexol**			
	**Plasma (** **μ** **mol/L)**		**Clearance (mL/min/1.73 m** ^**2**^ **)**		**Plasma clearance (mL/min/1.73 m** ^**2**^ **)**		**Renal clearance (mL/min/1.73 m** ^**2**^ **)**	
**Subject**	**A**	**B**	**A**	**B**	**A**	**B**	**A**	**B**
*Healthy Volunteers*								
1	59.7	65.7			101.9	107.4		
2	58.8	62.9			105.4	105.3		
3	52.3	61.0			100.7	102.6		
4	68.2	68.6			104.2	105.1		
5	75.0	75.5			119.3	127.7		
6	76.6	78.7	101.9	100.3	89.1	82.0	79.5	75.8
7	85.1	90.1	115.5	104.7	97.5	89.1	105.1	92.3
8	75.5	77.7	70.7	77.3	80.7	92.1		
9	55.3	57.9	120.6	112.1	83.2	83.2	78.8	87.0
10	69.6	71.4	98.7	101.0	77.9	89.4		
11	94.7	91.6	105.5	104.7	87.7	88.0	95.4	85.1
Mean ± SD	70.1 ± 13.0	72.9 ± 11.3	102.2 ± 17.5	100.0 ± 11.9	95.2 ± 12.6	97.4 ± 13.6	89.7 ± 12.8	85.1 ± 6.9
P value		P = 0.59		P = 0.46		P = 0.75		P = 0.54
*Chronic Kidney Disease*								
12	177.0	200.2	43.8	43.9	40.7	40.5	33.7	33.4
13	219.8	215.9	38.6	36.9	33.9	38.5	27.0	23.3
14	139.3	146.8	25.8	22.8	41.3	44.3		
15	118.3	126.5	78.6	77.2	61.2	62.2	53.5	50.8
16	100.0	108.0	40.7	46.9	51.7	52.9	38.8	37.6
17	243.5	253.9	26.6	23.8	28.3	31.7	26.0	18.6
Mean ± SD	166.3 ± 57.2	175.2 ± 57.2	42.4 ± 19.3	41.9 ± 20.0	42.8 ± 12.	45.0 ± 10.9	35.8 ± 11.2	32.7 ± 12.6
P value		P = 0.79		P = 0.77		P = 0.70		P = 0.70
*All volunteers*								
Mean ± SD	104.0 ± 58.1	109.0 ± 60.3	72.2 ± 35.8	70.9 ± 34.2	76.7 ± 28.5	78.9 ± 28.6	59.8 ± 30.5	56.0 ± 29.3
P value		P = 0.81		P = 0.93		P = 0.82		P = 0.79

GFR has been rounded to one decimal place. The student’s *t*-test was used to calculate P values comparing method A clearances with method B. CI = confidence interval, SD = standard deviation, HV = healthy volunteer, CKD = chronic kidney disease.

### Association, agreement and precision of GFR calculations

**Figure 2:**
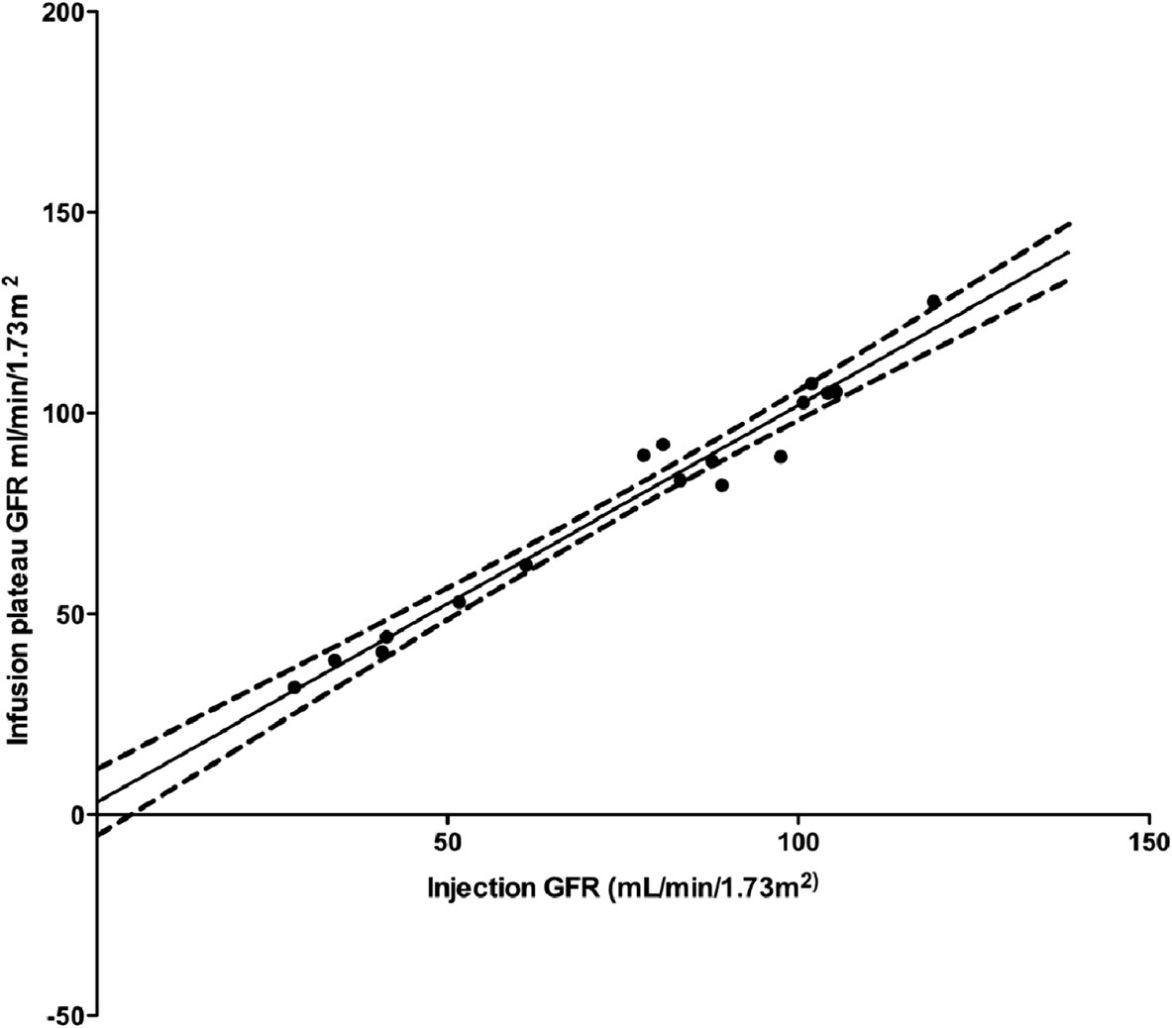
**Association between plasma clearance GFR calculated by the single bolus and the experimental continuous infusion of low dose Iohexol (CILDI).** Solid line = line of association, dashed lines = error margins of association line. Slope = 0.988 ± 0.048, Intercept = 3.08 ± 3.92, Pearson’s correlation, *r* = 0.983, p < 0.0001.

**Figure 3:**
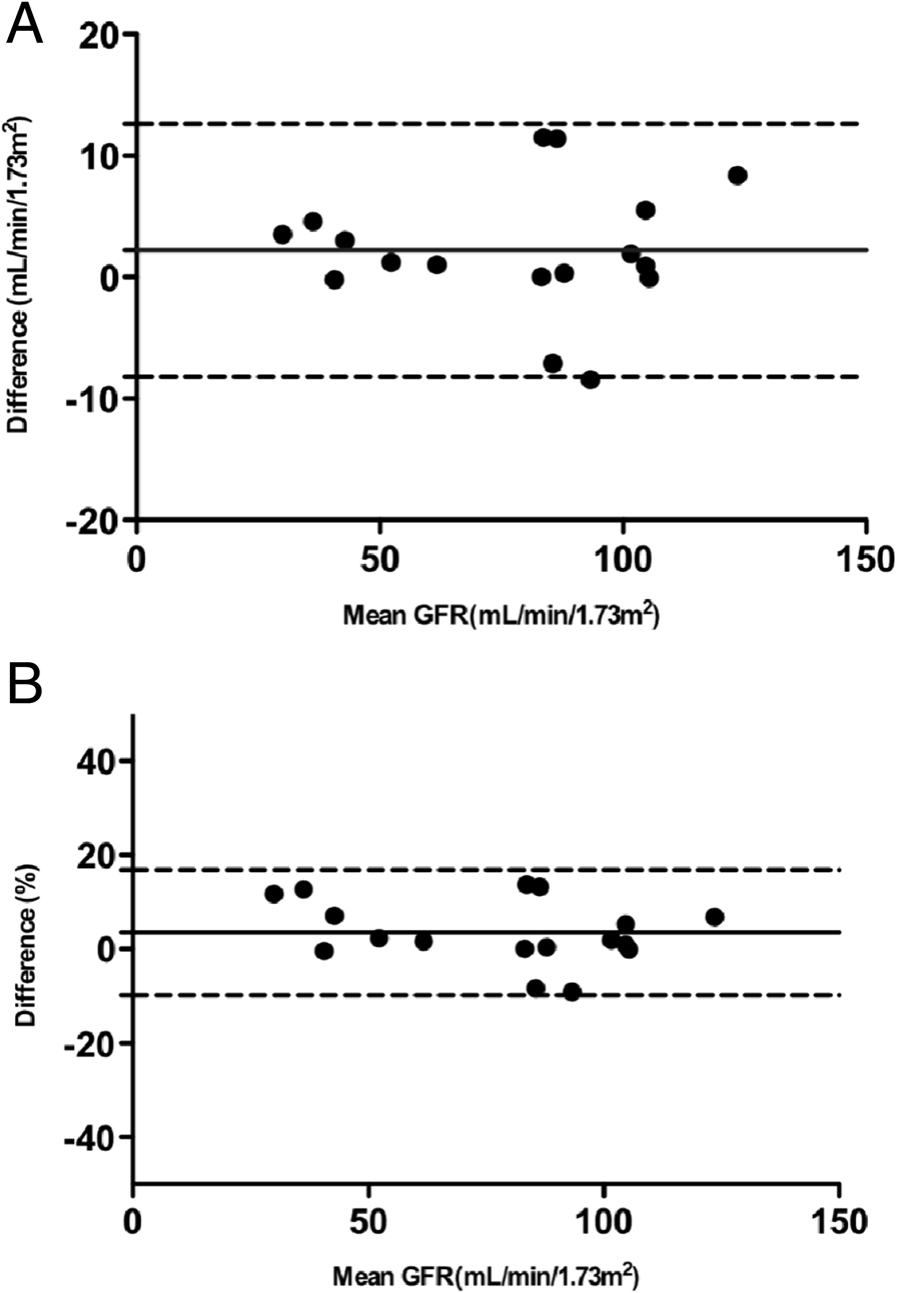
**Bland-Altman comparison of plasma clearance GFR by single Iohexol bolus method and the experimental method (CILDI).** Central solid lines are bias and outer dashed lines are limits of agreement, *n* = 17. **A)** Difference is measured in mL/min/1.73 m^2^. Bias = 2.2 mL/min/1.73 m^2^, SD of bias = 5.3 mL/min/1.73 m^2^, 95% limits of agreement from −8.2 to +12.6 mL/min/1.73 m^2^, **B)** Difference is measured as percentage difference in GFR. Bias = 3.5%, SD of bias = 6.8%, 95% limits of agreement from −9.8 to +16.8%.

There was no significant difference in PC between methods A (SBI) and B (CILDI) overall or on sub-group analysis; Table 3. Association (Figure 2) and agreement (Figure 3) between the methods were good. Sub-group analysis revealed closer limits of agreement, when measured in mL/min/1.73 m^2^, in the CKD group, although this was not significant when measured as percentage difference in GFR. When GFR was measured by CILDI, bias in the HV group was 2.2 mL/min/1.73 m^2^ (2.2%), limits of agreement −10.7 to 15.1 mL/min/1.73 m^2^ (−12.1 to 16.6%); in patients with CKD bias was 2.2 mL/min/1.73 m^2^ (5.8%), with limits of agreement −1.3 to 5.7 mL/min/1.73 m^2^ (−5.0 to 16.6%). Intra-individual variations in GFR and precision of GFR calculations are summarised in Table 4. Time to steady state (*Css)* was less than 10 h in all subjects (Table 4). The difference in GFR which depicts AKI can be determined by measuring the precision of CILDI at 95% and 99% confidence intervals and at 3 standard deviations (ie 99.7% confidence intervals). A difference of greater than 10.3% (3 standard deviations) after time to *Css* had elapsed, depicts a true difference in GFR (p < 0.003). The Pearson correlation between time to steady state concentration and GFR is 0.82.

**Table 4:** **Precision of GFR measurements and time to steady state during CILDI**

**Method B Precision (Plasma clearance)**				
**Subject**	**95% CI**	**99% CI**	**3 SD^**	**Time to steady state (min) [no. samples]**
*Healthy Volunteers*				
1	2.9	3.9	4.5	98 [7]
2	10.4	13.6	15.9	248 [4]
3	2.7	3.6	4.2	126 [6]
4	8.3	10.8	12.7	287 [4]
5	1.3	1.7	2.0	118 [7]
6	3.1	4.0	4.7	127 [6]
7	4.5	6.0	6.9	86 [8]
8	3.5	4.7	5.4	205 [5]
9	4.7	6.2	7.2	110 [7]
10	5.8	7.6	8.9	154 [6]
11	1.0	1.4	1.6	143 [6]
Mean ± SD	4.4 ± 2.9	5.8 ± 3.7	6.7 ± 4.4	155 ± 126
*Chronic Kidney Disease*				
12	6.6	8.7	10.2	555 [3*]
13	17.7	23.2	27.1	598 [3*]
14	15.8	20.8	24.2	508 [3*]
15	6.3	8.3	9.6	327 [4]
16	6.9	9.1	10.6	335 [3]
17	12.5	16.3	19.1	600 [3*]
Mean ± SD	11.0 ± 5.1	14.4 ± 6.6	16.8 ± 7.4	487 ± 65
*All Volunteers*				
Mean ± SD	6.7 ± 4.9	8.8 ± 6.4	10.3 ± 7.4	172 ± 185

Precision of individual GFR measurements was calculated for each subject during CILDI to confidence intervals (CI) of 95% and 99%, and to 3 standard deviations (SD). Precision results are expressed as percentages. ^3SD = 99.7%CI. Theoretical time to steady state (Css) was calculated by drawing a 2-phase exponential decay curve, using Graphpad Prism®, version 5.0d (Graphpad software, Inc.). All samples used after this time was achieved were used, except where Css was >8 h. In these circumstances, the last 3 samples were used (*).

### Plasma and renal clearance

**Figure 4:**
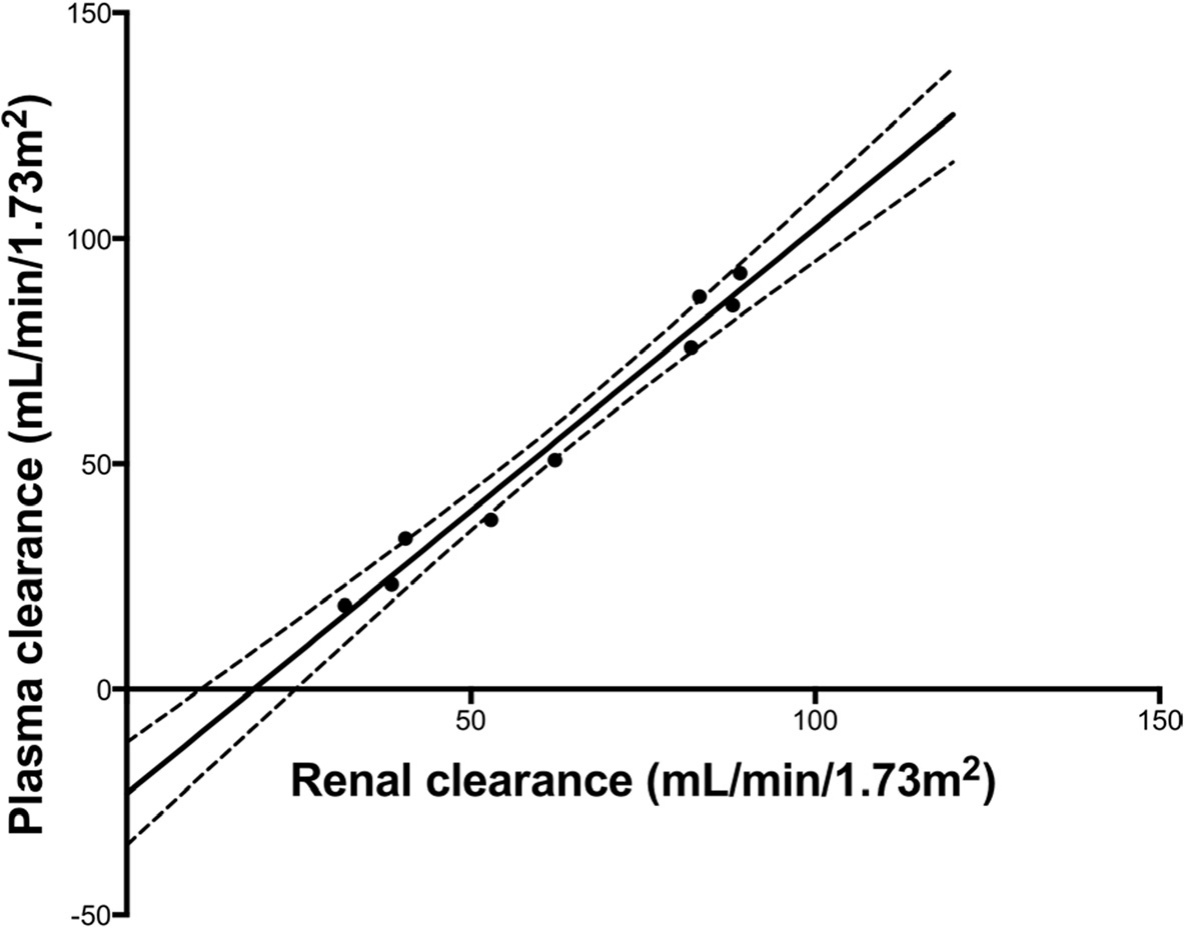
**Correlation between plasma and renal clearance during CILDI.** Solid line = line of association, dashed lines = error margins of association line. Pearson’s correlation (*r*) = 0.989, Intercept = −23.3 ± 4.8, slope = 1.25 ± 0.07.

**Figure 5:**
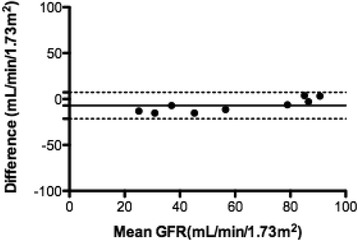
**Bland-Altman comparison of plasma clearance GFR and Renal clearance GFR during CILDI.** Difference is measured in mL/min/1.73 m^2^. Bias = −7.1 mL/min/1.73 m^2^, SD of bias = 7.3, 95% Limits of agreement = −21.5 to +7.2 mL/min/1.73 m^2^. *n* = 9. Although based on small numbers sub-group analysis suggests a smaller bias in the HV group. In the HV group bias was −0.5 mL/min/1.73 m^2^ (limits of agreement −10.0 to +8.9 mL/min/1.73 m^2^); in the CKD group bias was −12.4 mL/min/1.73 m^2^ (limits of agreement −19.0 to −5.8 mL/min/1.73 m^2^).

Post-micturition bladder scans revealed incomplete bladder voiding in 8 subjects; making RC difficult to perform accurately. Consequently, only 9 RC were performed satisfactorily. Although the correlation between PC and RC was 0.989 (Figure 4), measurement of GFR by PC overestimated RC of Iohexol by 7.1 ± 7.3 mL/min/1.73 m^2^ (Table 2; Figure 5).

### Assessment for crossover effects

The Kolgomorov-Smirnov test confirmed GFR measurements had Gaussian distributions during both methods, before and after logarithmic transformation of data. No period effect was observed using raw data (paired 2-tailed *t*-test; p = 0.85) or transformed data (paired 2-tailed *t*-test; p = 0.91) [26]. Pairing of raw and transformed data were matched (Pearson’s *r* =0.98, p < 0.0001).

### Futility analysis

An interim analysis was conducted after the first batch analysis of samples. The ratio of the means has a difference of 3.5% (95% CI: 0.998 to 1.071). A futility test was performed to determine whether mean GFR observed in both methods would differ by >10% if the trial continued until 30 subjects had been recruited. The limit of 10% is 5.8 standard errors beyond the observed difference of 3.5% and so a 10% difference is ruled out at p < 10^−7^.

## Discussion

### Summary of results

**Figure 6:**
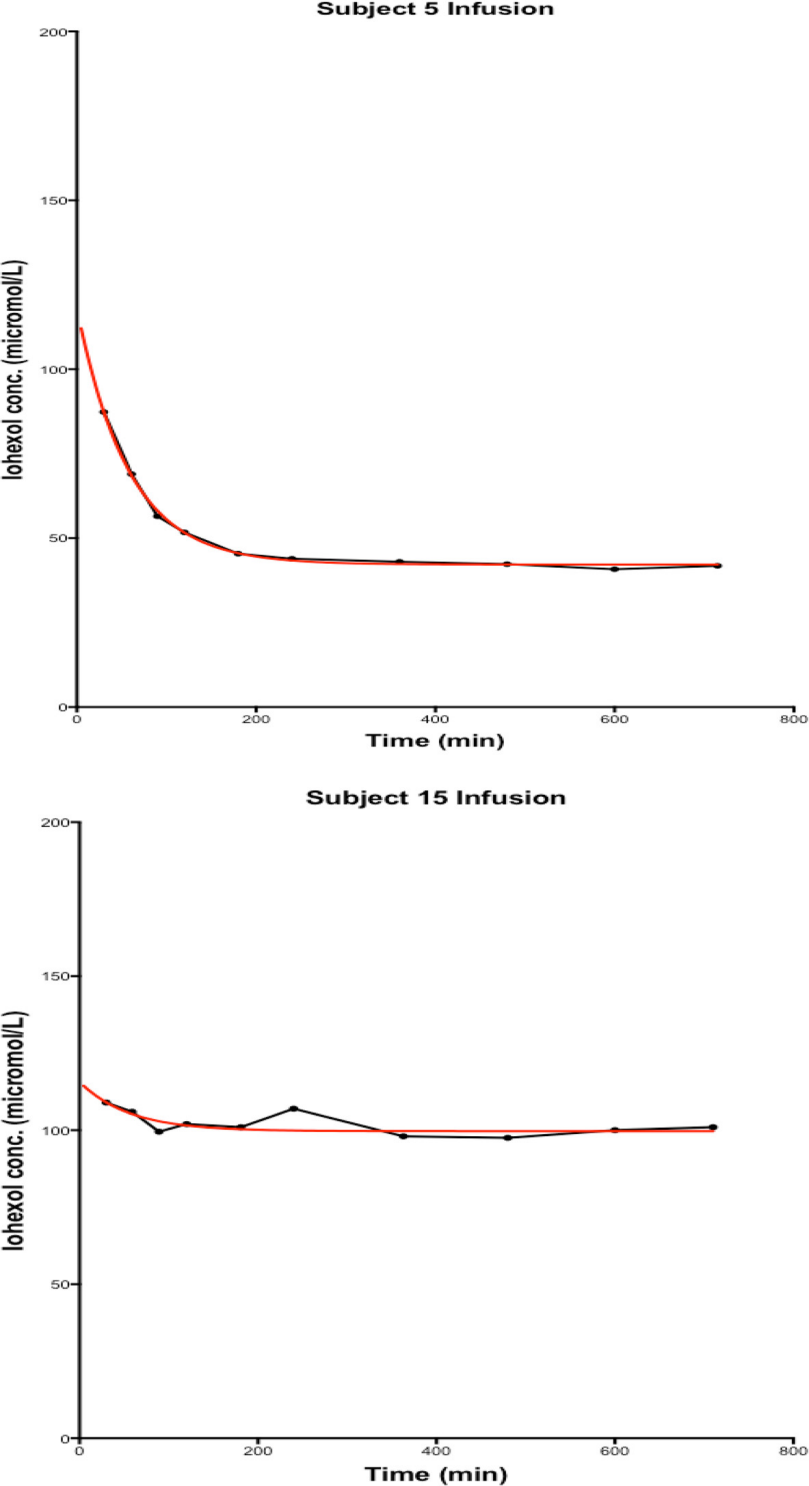
**Examples of Iohexol concentrations achieved during Method B (CILDI).** Different steady state concentrations were observed in subjects 5 and 15. GFR in subject 5 was 127.7 mL/min/1.73 m^2^, and GFR in subject 15 was 62.2 mL/min/1.73 m^2^. Concentrations and time were plotted on a 2-phase exponential decay curve, using Graphpad Prism®, version 5.0d (Graphpad software, Inc.). The black line connects the Iohexol concentrations, the red line depicts the steady state concentration.

We have demonstrated that measurement of the plasma clearance of CILDI is accurate and precise. There is an excellent correlation (Figure 2) with the plasma clearance of a single Iohexol bolus, and Bland Altman comparison [23] reveals a small bias with close limits of agreement (Figure 3). During CILDI, mean intra-individual variation in GFR was 10.3% (p < 0.003). Once the time to steady state concentration (*Css)* had elapsed, all subjects reached *Css* within 10 h. It is theoretically possible to determine change in GFR from single measurements of Iohexol made after 10 h: variations greater than 10.3% represent changing GFR. If these data were applicable in the context of AKI, this is significantly less than the 50% change in SCr needed to define AKI by current criteria [1-3]. Intra-individual fluctuations in GFR may be caused by differences in fluid balance throughout the day [27] and circadian rhythms [28]. Correlation and agreement between CILDI plasma and renal clearance, when measured, were also good (Figures 4 and 5). Figure 6 demonstrates an increased steady state concentration in CKD subject 15, compared with HV subject 5.

Iohexol has many properties of an “ideal GFR marker” [29]: it diffuses rapidly into the extracellular space; it undergoes less than 2% protein binding; over 99% is filtered at the glomerulus [16]; and it undergoes no renal tubular reabsorption or secretion. It has an excellent safety profile [30] and, when measured by high performance liquid chromatography / electrospray tandem mass spectrometry (LC-MSMS), gives highly reproducible results with low inter- and intra-patient coefficient of variation [31]. LC-MSMS has been validated to accurately and precisely measure plasma Iohexol concentrations less than 10 μmol/L, minimising volumes needed for administration [31]. Accuracy of bolus injection of Iohexol to measure GFR has been confirmed in studies comparing it with Inulin infusions [12], and it is substantially cheaper and more readily available than Inulin [32].

An infusion of Iohexol over 4 hours has previously been validated to measure GFR in subjects with normal renal function [10], however, we have modified this is 2 ways. First, we have employed a much lower dose of Iohexol. This has allowed us to increase the duration of the infusion to more than 72 hours, maintaining the total dose given well within safe limits. Secondly, we have also tested the method in patients with stable CKD with a wide range of GFR from normal to <30 mL/min/1.73 m^2^. Six of the subjects in our healthy volunteer category met criteria for CKD stage 2 [33]; the patient with the lowest GFR in the CKD group had CKD stage 4. If the time to *Css* were markedly increased in patients with CKD, then this would limit the applicability in patients with AKI. In our trial, time to *Css* was under 10 hours. We hope these modifications will allow us to apply this method in patients with AKI.

CILDI could potentially be continued for prolonged periods (e.g. up to 6 days), with regular plasma and urine measurements, allowing the course of moderate and slowly developing AKI to be monitored. It is also potentially useful in rapidly evolving AKI because Iohexol concentrations will continue to rise, rather than move towards the expected *Css*. Although time to *Css* is <10 h, subjects with known baseline GFR >60 mL/min/1.73 m^2^, will have time to *Css*155 ± 126 min (Table 4), thus increasing the potential applicability of CILDI to detect rapidly evolving AKI if *Css* is not achieved within this time. This also applies to SCr, however, CILDI has theoretical advantages over SCr because the production rate of creatinine is often reduced in critically ill patients with AKI [7]. In addition, LC-MSMS measurements of Iohexol concentration are more accurate than routine laboratory SCr measurements.

### Limitations

Our data are limited to a small number of subjects with stable GFR and *Vd*. Although the subjects in our trial may differ from patients with AKI, this study lays the groundwork for studies in patients with AKI, providing an evidence base that CILDI may be preferable to SCr as a research tool. Patients with AKI are unlikely to reach equilibrium, however, it will theoretically be possible to measure changes in GFR occurring after time to *Css*, or, in rapidly developing AKI occurring before time to *Css*, to demonstrate increasing plasma concentrations, rather than the expected approach towards *Css*. The natural history of AKI and the timing of when its associated pathophysiological changes occur are unknown. Although CILDI detects smaller changes in GFR than needed in SCr to define AKI, it is possible that the pathophysiological effects may occur at changes too small to detect with CILDI. Furthermore, they may occur before time to *Css*. A presumption in using CILDI is that the baseline eGFR is known, or at least >23 mL/min/1.73 m^2^, and hence CILDI may not be suitable for all patients, or it may not be reliable once GFR drops to very low values. In most cases of AKI, however, we anticipate that fluctuations in *Css* will be observed with CILDI.

LC-MSMS allows quantification of relatively low concentrations of Iohexol, but is not readily available and requires time and expertise to obtain results. This method is, therefore, not currently suitable for routine clinical use; it is primarily useful as a research tool to measure evolving GFR in AKI so that pathophysiological effects can be measured and timed accurately. It will also allow monitoring of the natural history of AKI in specific disease states (e.g. sepsis, post nephrectomy, following major surgery). Other Iohexol GFR techniques (e.g. blotting) require higher concentrations of Iohexol than CILDI and pre-dispose towards toxicity. LC-MSMS is required, however, because of its specificity. It is possible that other laboratories may have a precision that is different from 10.3% at 3 standard deviations, so this study needs external validation; the co-efficient of variation in our Iohexol measurements, is, however, similar to that in the literature [31].

Plasma clearance of CILDI overestimated renal clearance by 7.1 ± 7.3 mL/min/1.73 m^2^ (Figure 5). This is, however, less than the difference of approximately 10 mL/min/1.73 m^2^ observed in studies measuring GFR with Inulin and EDTA [34,35]. The difference between renal clearance and plasma clearance was more apparent in subjects with lower GFR. Our study demonstrates that, even under experimental conditions, “gold standard” urine collection and bladder voiding are often incomplete without urinary catheterisation. This limits precision of measurements; GFR measurement with urine collection is therefore not sufficiently robust for routine clinical application. Three patients were excluded due to subcutaneous injection when administered via peripheral cannulae; it may be more appropriate to administer CILDI via central venous catheters in clinical settings.

Radio-contrast agents have been associated with AKI [36], although recent meta-analyses [37,38] show no difference in the incidence of AKI occurring in patients receiving contrast and matched controls without contrast. This implies that AKI occurring after contrast administration is likely to be multi-factorial and may be more attributable to the underlying illness than contrast media. This particular aspect of AKI aetiology is the subject of on going debate within critical care societies and warrants its own detailed investigation before final conclusions can be made. Proponents suggest a threshold ratio of iodinated contrast volume to weight and baseline renal function that has to be exceeded before contrast-associated AKI develops [39]. If CILDI were continued for 72 h, the volume of Iohexol used is less than half this ratio for an adult weighing 40Kg and, if all the Iohexol accumulated, would take a minimum of 6 days before this threshold was exceeded. Conversely, bolus Iohexol injection [9] would require repeated administration of larger volumes of Iohexol to measure changing GFR in critically ill patients and the threshold for toxicity would be exceeded much sooner. In addition, the washout period required makes bolus methods unsuitable for use in AKI.

Confounding factors will undoubtedly emerge when the method is applied to acutely unwell patients, who will be unstable with changing parameters of GFR and *Vd*. However, the CILDI method is simple to apply and will enable the measurement of both plasma and renal clearance of Iohexol in critically ill patients, thus providing a measure of dynamic changes in GFR and allowing the direct investigation in vivo of the impact of physiological and pathological perturbations on renal function for the first time.

## Conclusion

We have developed a tool for measuring GFR in stable populations that is now ready to be tested in patients at risk of AKI. In our trial, all subjects achieved a steady-state concentration within 10 h. Measurements made after this time in critically ill patients that change by more than 10.3% likely represent changing GFR and depict evolving AKI. The next stage is to investigate the applicability of CILDI in patients with AKI. We hypothesise that CILDI may be a more sensitive method of detecting and monitoring AKI than changes in SCr and if proven to be so, may provide a new standard to which other methods of measuring GFR in AKI are compared.

### Key messages

- GFR can be accurately and precisely measured by CILDI.
- GFR that varies by >10.3%, using CILDI, represents AKI (p < 0.003).
- Time to steady state in subjects with GFR >28 mL/min/1.73 m^2^ is <10 hours in all subjects.
- CILDI is now ready to be investigated in patients with AKI and at risk of AKI.

## Abbreviations

AKI: Acute kidney injury
BSA: Body surface area
CI: Confidence interval
CILDI: Continuous infusion of low dose Iohexol
CKD: Chronic kidney disease
Css: Steady state concentration
CV: Co-efficient of variation
EDTA: Ethylene diamine tetraacetic acid
GFR: Glomerular filtration rate
HV: Healthy volunteer
LC-MSMS: High performance liquid chromatography / electrospray tandem mass spectrometry
LD: Loading dose
MDRD: Modification of diet in renal disease formula
*MInf*: Mass of substance infused per unit time
PC: Plasma clearance
RC: Renal clearance
SBI: Single bolus injection [of Iohexol]
SCr: Serum creatinine concentration
UO: Urine output
*Vd*: Volume of distribution

## References

[1] Mehta R, Kellum JA, Shah SV, Molitoris BA, Ronco C, Warnock DG (2007). Acute Kidney Injury Network: report of an initiative to improve outcomes in acute kidney injury. Crit Care.

[2] Bellomo R, Ronco C, Kellum JA, Mehta RL, Palevsky P, Acute Dialysis Quality Initiative workgroup (2004). Acute renal failure – definition, outcome measures, animal models, fluid therapy and information technology needs: the Second International Consensus Conference of the Acute Dialysis Quality Initiative (ADQI) Group. Crit Care.

[3] Kidney Disease Improving Global Outcomes work group (2012). KDIGO Clinical Practice Guidelines for Acute Kidney Injury. Kidney Int Suppl.

[4] Ostermann M, Chang RWS (2011). Challenges of defining acute kidney injury. Q J Med.

[5] Waikar SS, Bonventre JV (2009). Creatinine kinetics and the definition of acute kidney injury. J Am Soc Nephrol.

[6] Doi K, Yuen PS, Eisner C, Hu X, Leelahavanichkul A, Schnermann J (2009). Reduced production of creatinine limits its use as marker of kidney injury in sepsis. J Am Soc Nephrol.

[7] Wilson FP, Sheehan JM, Mariani LH, Berns JS (2012). Creatinine generation is reduced in patients requiring continuous venovenous hemodialysis and independently predicts mortality. Nephrol Dial Transplant.

[8] Coca SG, Yalavarthy R, Concato J, Parikh CR (2008). Biomarkers for the diagnosis and risk stratification of acute kidney injury: a systematic review. Kidney Int.

[9] Erley CM, Badar BD, Berger ED, Vochazer A, Jorzik JJ, Dietz K (2001). Plasma clearance of iodine contrast media as a measure of glomerular filtration rate in critically ill patients. Crit Care Med.

[10] Sterner G, Frennby B, Mansson S, Nyman U, Van Western D, Almén T (2008). Determining “true” glomerular filtration rate in healthy adults using infusion of inulin and comparing it with values obtained using other clearance techniques or prediction equations. Scand J Urol Nephrol.

[11] Gaspari F, Perico N, Ruggenenti P, Mosconi L, Amuchastegui CS, Guerini E (1995). Plasma clearance of nonradioactive iohexol as a measure of glomerular filtration rate. J Am Soc Nephrol.

[12] Effersöe H, Groth S, Jensen LI, Golman K (1990). Measurement of renal function with iohexol. A comparison of iohexol, 99mTc-DTPA and 51Cr-EDTA clearance. Invest Radiol.

[13] Levey AS, Stevens LA, Schmid CH, Zhang YL, Castro AF, Feldman HI (2009). CKD-EPI (Chronic Kidney Disease Epidemiology Collaboration). Ann Intern Med.

[14] World Medical Association Declaration of Helsinki. Ethical Principles for Medical Research Involving Human Subjects. 59th WMA General Assembly, Seoul, October 2008. http://www.wma.net/en/30publications/10policies/b3/17c.pdf.

[15] European medicines agency. ICH Topic E 6 (R1) Guideline for Good Clinical Practice. http://www.emea.europa.eu/docs/en_GB/document_library/Scientific_guideline/2009/09/WC500002874.pdf.

[16] GE Healthcare Inc (2009). OMNIPAQUE (Iohexol): Summary of product characteristics.

[17] Hemmegarn BR, Zhang J, Manns BJ, Tonelli M, Larsen E, Ghali WA (2006). Progression of kidney dysfunction in the community-dwelling elderly. Kidney Int.

[18] Mosteller RD (1987). Letters to editor. N Engl J Med.

[19] Product Information for Agilia MC Injectomat pump. Accessed online via URL http://www.fresenius-kabi.com (accessed 4 November 2010)

[20] Brochner-Mortensen J (1972). A simple method for the determination of Glomerular Filtration Rate. Scand J Clin Lab Invest.

[21] Kilbride HS, Stevens PE, Eaglestone G, Knight S, Carter JL, Delaney MP (2012). Accuracy of the MDRD (Modification of diet in Renal Disease) Study and CKD-EPI (CKD Epidemiology Collaboration) Equations for Estimation of GFR in the elderly. Am J Kidney Dis.

[22] Shannon JA (1935). The renal excretion of creatinine in man. J Clin Invest.

[23] Bland JM, Altman DG (1999). Measuring agreement in medical comparison studies. Stat Methods Med Res.

[24] Fleming JS, Zivanovic MA, Blake GM, Burniston M, Cosgriff PS. Guidelines for the Measurement of Glomerular Filtration Rate using Plasma Sampling. http://www.bnms.org.uk (accessed 23 December 2014). 10.1097/01.mnm.0000136715.71820.4a 15266169

[25] James TJ, Lewis AV, Tan GD, Altmann P, Taylor RP, Levy JC (2007). Validity of simplified protocols to estimate glomerular filtration rate using iohexol clearance. Ann Clin Biochem.

[26] Mills M, Armitage P (1979). The two-period cross-over clinical trial. B J Clin Pharm.

[27] van Acker BAC, Koomen GCM, Arisz L (1995). Drawbacks of the constant-infusion technique for measurement of renal function. Am J Physiol.

[28] Koopman MG, Koomen GCM, Krediet E, de Moor EAM, Hoek FJ, Arisz L (1989). Circadian rhythm of glomerular filtration rate in normal individuals. Clin Sci.

[29] Olsson B, Aulie Å, Sveen K, Andrew E (1983). Human Pharmacokinetics of Iohexol A New Nonionic Contrast Medium. Invest Radiol.

[30] Lundqvist S, Holmberg G, Jakobsson G, Lithner F, Skinningsrud K, Stegmayr B (1998). Assessment of possible nephrotoxicty from Iohexol in patients with normal and impaired renal function. Acta Radiol.

[31] Cavalier E, Rozet E, Dubois N, Charlier C, Hubert P, Chapelle J-P (2008). Performance of Iohexol determination in serum and urine by HPLC: validation, risk and uncertainty assessment. Clinica Chem Acta.

[32] Kays AJ (2008). Economics. Biology and Chemistry of Jerusalem Artichoke: Helicanthus tuberosus L.

[33] Nissenson AR, Pereira BJG, Collins AJ, Steinberg EP (2001). Prevalence and characteristics of individuals with chronic kidney disease in a large health organization. Am J Kid Dis.

[34] Schnurr E, Lahme W, Küppers H (1980). Measurement of renal clearance of inulin and PAH in the steady state without urine collection. Clin Nephrol.

[35] Moore AEB, Park-Holohan S-J, Blake GM, Fogelman I (2003). Conventional measurements of GFR using Cr-51-EDTA overestimate true renal clearance by 10%. Eur J Nucl Med Mol Imaging.

[36] Barrett BJ, Carlisle EJ (1993). Meta-analysis of the relative nephrotoxicity of high- and low-osmolality iodinated contrast media. Radiology.

[37] McDonald RJ, McDonald JS, Bida JP, Carter RE, Fleming CJ, Misra S (2013). Intravenous contrast material-induced nephropathy: causal or coincident phenomenon. Radiol.

[38] McDonald JS, McDonald RJ, Comin J, Williamson EE, Katzberg RW, Murad MH (2013). Frequency of acute kidney injury following intravenous contrast medium administration: a systematic review and meta-analysis. Radiol.

[39] Brown JR, Robb JF, Block CA, Schoolwerth AC, Kaplan AV, O’Connor GT (2010). Does safe dosing of iodinated contrast prevent contrast-induced acute kidney injury?. Circ Cardiovasc Interv.

